# Formal guidelines: management of acute respiratory distress syndrome

**DOI:** 10.1186/s13613-019-0540-9

**Published:** 2019-06-13

**Authors:** Laurent Papazian, Cécile Aubron, Laurent Brochard, Jean-Daniel Chiche, Alain Combes, Didier Dreyfuss, Jean-Marie Forel, Claude Guérin, Samir Jaber, Armand Mekontso-Dessap, Alain Mercat, Jean-Christophe Richard, Damien Roux, Antoine Vieillard-Baron, Henri Faure

**Affiliations:** 1 Service de Médecine Intensive - Réanimation, Hôpital Nord, Chemin des Bourrely, 13015 Marseille, France; 2 Medical Intensive Care Unit, Centre Hospitalier Régional et Universitaire de Brest, site La Cavale Blanche, Bvd Tanguy Prigent, 29609 Brest Cedex, France; 3 Interdepartmental Division of Critical Care Medicine, University of Toronto, Toronto, Canada; 4 Service de Médecine Intensive - Réanimation, Hôpital Cochin, Hôpitaux Universitaires Paris-Centre, Assistance Publique - Hôpitaux de Paris, 27 Rue du Faubourg Saint-Jacques, 75014 Paris, France; 5 Service de Réanimation, Institut de Cardiologie, Groupe Hospitalier Pitié– Salpêtrière, Assistance Publique–Hôpitaux de Paris, 47, boulevard de l’Hôpital, 75013 Paris, France; 6 Intensive Care Unit, Louis Mourier Hospital, AP-HP, 178 Rue des Renouillers, 92700 Colombes, France; 7 Service de Réanimation Médicale, Hôpital De La Croix Rousse, Hospices Civils de Lyon, 103 Grande Rue de la Croix Rousse, 69004 Lyon, France; 8 Department of Anesthesiology and Intensive Care (DAR B), Saint Eloi University Hospital, Montpellier, France; 9 Service de Réanimation Médicale, Hôpitaux Universitaires Henri-Mondor, AP-HP, DHU A-TVB, 94010 Créteil, France; 10 Medical Intensive Care Department, Angers University Hospital, 4, rue Larrey, 49933 Angers Cedex, France; 11 Emergency Department, General Hospital of Annecy, Annecy, France; 12 Hospital Ambroise Paré, Assistance Publique-Hôpitaux de Paris, Boulogne, France; 13 Service de Médecine Intensive - Réanimation, Centre Hospitalier Intercommunal Robert Ballanger, 93602 Aulnay-sous-Bois, France

## Abstract

Fifteen recommendations and a therapeutic algorithm regarding the management of acute respiratory distress syndrome (ARDS) at the early phase in adults are proposed. The Grade of Recommendation Assessment, Development and Evaluation (GRADE) methodology has been followed. Four recommendations (low tidal volume, plateau pressure limitation, no oscillatory ventilation, and prone position) had a high level of proof (GRADE 1 + or 1 −); four (high positive end-expiratory pressure [PEEP] in moderate and severe ARDS, muscle relaxants, recruitment maneuvers, and venovenous extracorporeal membrane oxygenation [ECMO]) a low level of proof (GRADE 2 + or 2 −); seven (surveillance, tidal volume for non ARDS mechanically ventilated patients, tidal volume limitation in the presence of low plateau pressure, PEEP > 5 cmH2O, high PEEP in the absence of deleterious effect, pressure mode allowing spontaneous ventilation after the acute phase, and nitric oxide) corresponded to a level of proof that did not allow use of the GRADE classification and were expert opinions. Lastly, for three aspects of ARDS management (driving pressure, early spontaneous ventilation, and extracorporeal carbon dioxide removal), the experts concluded that no sound recommendation was possible given current knowledge. The recommendations and the therapeutic algorithm were approved by the experts with strong agreement.

## Introduction

Acute respiratory distress syndrome (ARDS) is an inflammatory process in the lungs that induces non-hydrostatic protein-rich pulmonary oedema. The immediate consequences are profound hypoxemia, decreased lung compliance, and increased intrapulmonary shunt and dead space. The clinicopathological aspects include severe inflammatory injury to the alveolar-capillary barrier, surfactant depletion, and loss of aerated lung tissue.

The most recent definition of ARDS, the Berlin definition, was proposed by a working group under the aegis of the European Society of Intensive Care Medicine [1]. It defines ARDS by the presence within 7 days of a known clinical insult or new or worsening respiratory symptoms of a combination of acute hypoxemia (PaO_2_/FiO_2_ ≤ 300 mmHg), in a ventilated patient with a positive end-expiratory pressure (PEEP) of at least 5 cmH_2_O, and bilateral opacities not fully explained by heart failure or volume overload. The Berlin definition uses the PaO_2_/FiO_2_ ratio to distinguish mild ARDS (200 < PaO_2_/FiO_2_ ≤ 300 mmHg), moderate ARDS (100 < PaO_2_/FiO_2_ ≤ 200 mmHg), and severe ARDS (PaO_2_/FiO_2_ ≤ 100 mmHg).

Much information on the epidemiology of ARDS has accrued from LUNG SAFE, an international, multicenter, prospective study conducted in over 29,000 patients in 50 countries [2]. During this study, ARDS accounted for 10% of admissions to intensive care unit (ICU) and 23% of ventilated patients. Hospital mortality, which increased with the severity of ARDS [2], was about 40%, and reached 45% in patients presenting with severe ARDS [2–4]. Significant physical, psychological, and cognitive sequelae, with a marked impact on quality of life, have been reported up to 5 years after ARDS [5].

One of the most important results of the LUNG SAFE study was that ARDS was not identified as such by the primary care clinician in almost 40% of cases [2]. This was particularly so for mild ARDS, in which only 51% of cases were identified [2]. When all ARDS criteria were met, only 34% of ARDS patients were identified, suggesting that there was a delay in adapting the treatment, in particular mechanical ventilation [2]. This is the main reason why these formal guidelines are not limited to patients presenting with severe ARDS, but are intended for application to all mechanically ventilated intensive care patients.

Results from the LUNG SAFE study suggest that the ventilator settings used did not fully respect the principles of protective mechanical ventilation [2]. Plateau pressure was measured in only 40% of ARDS patients [2]. And only two-thirds of patients for whom plateau pressure was reported were receiving protective mechanical ventilation (tidal volume ≤ 8 mL/kg predicted body weight [PBW] and plateau pressure ≤ 30 cmH_2_O) [2]. Analysis of the LUNG SAFE results also shows a lack of relation between PEEP and the PaO_2_/FIO_2_ ratio [2]. In contrast, there was an inverse relation between FIO_2_ and SpO_2_, suggesting that the clinicians used FIO_2_ to treat hypoxemia. Lastly, prone positioning was used in just 8% of patients presenting with ARDS, essentially as salvage treatment [2].

The reduction in mortality associated with ARDS over the last 20 years seems to be explained largely by a decrease in ventilator-induced lung injury (VILI). VILI is essentially related to volutrauma closely associated with “strain” and “stress”. Lung stress corresponds to transpulmonary pressure (alveolar pressure–pleural pressure), and lung strain refers to the change in lung volume indexed to functional residual capacity of the ARDS lung at zero PEEP. So, volutrauma corresponds to generalized excess stress and strain on the injured lung [6–8]. High-quality CT scan studies and physiological studies have revealed that lung lesions are unequally distributed, the injury or atelectasis coexisting with aerated alveoli of close-to-normal structure [9]. ARDS is not a disease; it is a syndrome defined by a numerous clinical and physiological criteria. It is therefore not surprising that lung-protective ventilatory strategies that are based on underlying physiological principles have been shown to be effective in improving outcome. Minimizing VILI thus generally aims reducing volutrauma (reduction in global stress and strain). Lowering airway pressures has the theoretical dual benefit of minimizing overdistension of the aerated areas and mitigating negative hemodynamic consequences.

The current SRLF guidelines are more than 20 years old and so there was a pressing need to update them. The main aim with these formal guidelines was voluntarily to limit the topics to the best studied fields, so as to provide practitioners with solid guidelines with a high level of agreement between experts. Certain very important aspects of ARDS management were deliberately not addressed because there is insufficient assessment of their effects on prognosis (respiratory rate, mechanical power, target oxygenation, pH, PaCO_2_…). We also limited these guidelines to adult patients, to early phase of ARDS (first few days), and to invasive mechanical ventilation.

## Methods

These guidelines have been formulated by an expert working group selected by the SRLF. The organizing committee first defined the questions to be addressed and then designated the experts in charge of each question. The questions were formulated according to a Patient Intervention Comparison Outcome (PICO) format after a first meeting of the expert group. The literature was analyzed using Grade of Recommendation Assessment, Development and Evaluation (GRADE) methodology. A level of proof was defined for each bibliographic reference cited as a function of the type of study and its methodological quality. An overall level of proof was determined for each endpoint. The experts then formulated guidelines according to the GRADE methodology (Table 1).

**Table 1:** Recommendations according to the GRADE methodology

	Recommendations according to the GRADE methodology	
High level of proof	Strong recommendation “…should be done…”	Grade 1 +
Moderate level of proof	Optional recommendation “… should probably be done…”	Grade 2 +
Insufficient level of proof	Recommendation in the form of an expert opinion “The experts suggest…”	Expert opinion
Moderate level of proof	Optional recommendation “… should probably not be done…”	Grade 2 −
High level of proof	Strong recommendation “…should not be done…”	Grade 1 −
Insufficient level of proof		No recommendation

A high overall level of proof enabled formulation of a “strong” recommendation (should be done… GRADE 1 +, should not be done… GRADE 1 −). A moderate, low, or very low overall level of proof led to the drawing up of an “optional” recommendation (should probably be done… GRADE 2 +, should probably not be done… GRADE 2 −). When the literature was inexistent or insufficient, the question could be the subject of a recommendation in the form of an expert opinion (the experts suggest…). The proposed recommendations were presented and discussed at a second meeting of the expert group. Each expert then reviewed and rated each recommendation using a scale of 1 (complete disagreement) to 9 (complete agreement). The collective rating was done using a GRADE grid methodology. To approve a recommendation regarding a criterion, at least 50% of the experts had to agree and less than 20% had to disagree. For a strong agreement, at least 70% of the experts had to agree. In the absence of strong agreement, the recommendations were reformulated and rated again, with a view to reaching a consensus (Table 2).

**Table 2:** Summary of guidelines

	Recommendation	Level of proof
*Evaluation of ARDS management*		
R1.1	The experts suggest that the efficacy and safety of all ventilation parameters and therapeutics associated with ARDS management should be evaluated at least every 24 h	Expert opinion
*Tidal volume adjustment*		
R2.1.1	A tidal volume around 6 mL/kg of predicted body weight (PBW) should be used as a first approach in patients with recognized ARDS, in the absence of severe metabolic acidosis, including those with mild ARDS, to reduce mortality	Grade 1 +
R2.1.2	The experts suggest a similar approach for all patients on invasive mechanical ventilation and under sedation in ICU, given the high rate of failure to recognize ARDS and the importance of rapidly implementing pulmonary protection	Expert opinion
*Plateau pressure*		
R2.2.1	Once tidal volume is set to around 6 mL/kg predicted body weight, plateau pressure should be monitored continuously and should not exceed 30 cmH_2_O to reduce mortality	Grade 1 +
R2.2.2	The experts suggest that tidal volume should not be increased when the plateau pressure is well below 30 cmH_2_O, except in cases of marked, persistent hypercapnia despite reduction in instrumental dead space and increase of respiratory rate	Expert opinion
*Driving pressure*		
R2.3	Available data do not allow a recommendation to be made regarding respirator settings based solely on limitation of driving pressure. This limitation can be envisaged as a complement to limitation of plateau pressure in some special instances	No recommendation
*Positive end*-*expiratory pressure*		
R3.1.1	PEEP is an essential component of the management of ARDS and the experts suggest using a value above 5 cmH_2_O in all patients presenting with ARDS	Expert opinion
R3.1.2	High PEEP should probably be used in patients with moderate or severe ARDS, but not in patients with mild ARDS	Grade 2 +
R3.1.3	The experts suggest reserving high PEEP for patients in whom it improves oxygenation without marked deterioration of respiratory system compliance or hemodynamic status. PEEP settings should be individualized	Expert opinion
*High*-*frequency oscillation ventilation*		
R3.2	High-frequency oscillation ventilation should not be used in ARDS patients	Grade 1 −
*Recruitment maneuvers*		
R3.3	Recruitment maneuvers should probably not be used routinely in ARDS patients	Grade 2 −
*Early and short neuromuscular blockade*		
R4.1	A neuromuscular blocking agent should probably be considered in ARDS patients with a PaO_2_/FiO_2_ ratio < 150 mmHg to reduce mortality. The neuromuscular blocking agent should be administered by continuous infusion early (within 48 h after the start of ARDS), for no more than 48 h, with at least daily evaluation	Grade 2 +
*Early spontaneous ventilation*		
R4.2.1	Available data do not allow a recommendation to be made regarding a strategy of routine spontaneous ventilation in the acute phase of ARDS	No recommendation
R4.2.2	After the acute phase of ARDS, the experts suggest that ventilation with a pressure mode allowing spontaneous ventilation can be used when ensuring that the tidal volume generated is close to 6 mL/kg PBW and does not exceed 8 mL/kg PBW	Expert opinion
*Prone positioning*		
R5.1	Prone positioning should be used in ARDS patients with PaO_2_/FIO_2_ ratio < 150 mmHg to reduce mortality. Sessions of at least 16 consecutive hours should be performed	Grade 1 +
*Venovenous extracorporeal membrane oxygenation*		
R6.1	Venovenous extracorporeal membrane oxygenation (ECMO) should probably be considered in cases of severe ARDS with PaO_2_/FiO_2_ < 80 mmHg and/or when mechanical ventilation becomes dangerous because of the increase in plateau pressure and despite optimization of ARDS management including high PEEP, neuromuscular blocking agents, and prone positioning. The decision to use ECMO should be evaluated early by means of contact with an expert center	Grade 2 +
*Low*-*flow extracorporeal CO*_*2*_ *removal*		
R6.2	Available data do not allow a recommendation to be made concerning the use of low-flow extracorporeal CO_2_ removal during ARDS	No recommendation
*Inhaled nitrogen monoxide*		
R7.1	The experts suggest that inhaled nitric oxide can be used in cases of ARDS with deep hypoxemia, despite the implementation of a protective ventilation strategy and prone positioning and before envisaging use of venovenous ECMO	Expert opinion

### Area 1: Evaluation of ARDS management

**R1.1 - The experts suggest that the efficacy and safety of all ventilation parameters and therapeutics associated with ARDS management should be evaluated at least every 24** **h.**

**EXPERT OPINION**

**Rationale:**

Evaluation of the efficacy and safety of mechanical ventilation settings and treatments is a cornerstone of the early phase of the management of ARDS patients. As shown in these formal guidelines, the settings of ventilation parameters, such as PEEP, are based on their efficacy and tolerance. Moreover, the indication for some treatments depends on the severity of ARDS and these treatments will only be implemented when there is insufficient response to first-line treatments.

**Fig. 1:**
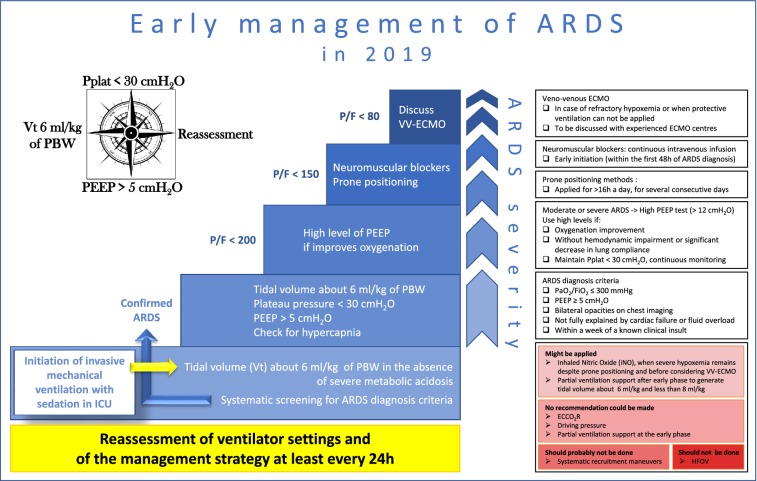
Therapeutic algorithm regarding early ARDS management (EXPERT OPINION)

Figure 1 shows the treatments implemented to patients with ARDS based on the severity of respiratory distress. The decision to initiate some treatments is taken after a “stabilization” phase [10] that includes optimization of mechanical ventilation as the first step of management. Early evaluation of efficacy based on the PaO_2_/FiO_2_ ratio is necessary in order to discuss the relevance of neuromuscular blocking agents and of prone positioning (Fig. 1).

The safety of drug therapies and procedures must also be regularly evaluated. These guidelines also address the main safety problems of the treatments. Literature support for such practices is lacking, and they are guided by good clinical sense.

Indeed, data are scarce on the benefits of regular assessment of ventilation settings and/or disease severity in ARDS patients. A single-center observational study has shown the value of systematic evaluation of respiratory mechanics during ARDS in the initial phase (mostly in the first 48 h) [11]. In this study, evaluation of the passive mechanics of the lung and thoracic cage, of the response to PEEP, and of alveolar recruitment prompted changes in ventilation parameters in most patients (41 of 61 analyzed). These changes were associated with improvements in plateau pressure (− 2 cmH_2_O on average), driving pressure (− 3 cmH_2_O on average), and oxygenation index [11].

It is difficult to define how often to assess ventilation parameters and treatments in ARDS. It seems that a frequency at least similar to that proposed for the evaluation of criteria for weaning from the ventilator (i.e. daily) is reasonable [12]. Nonetheless, more frequent assessment might be necessary and benefit in some cases.

### Area 2: Tidal volume management

Tidal volume adjustment

**R2.1.1 – A tidal volume around 6** **mL/kg of predicted body weight (PBW) should be used as a first approach in patients with recognized ARDS, in the absence of severe metabolic acidosis, including those with mild ARDS, to reduce mortality.**

**GRADE 1** **+, STRONG AGREEMENT**

**R2.1.2 – The experts suggest a similar approach for all patients on invasive mechanical ventilation and under sedation in ICU, given the high rate of failure to recognize ARDS and the importance of rapidly implementing pulmonary protection.**

**EXPERT OPINION**

**Rationale:**

To control potentially deleterious increases in PaCO_2_ (which raise pulmonary arterial pressure), a relatively high respiratory rate of between 25 and 30 cycles/min should be adopted first. Too high a rate, however, engenders a risk of dynamic hyperinflation and also increases each minute cumulative exposure to potentially risky insufflation. A PaCO_2_ below 50 mmHg is generally acceptable. A reduction in instrumental dead space is also appropriate, and a heated humidifier should be used in first intention.

The PBW should be calculated for each patient upon admission as a function of height and sex.

The tidal volume delivered will induce a pressure increase from the PEEP, thus necessitating monitoring of plateau pressure, which should be kept below 30 cmH_2_O.

Clinicians need to be aware of the potential risks of low tidal volume, such as dyssynchrony and double triggering. Guidelines on pressure and volume reduction issued in the late 1980s were based on experimental and clinical data [13–16]. Several randomized clinical trials with rather few subjects in the 1990s found no survival advantage of low tidal volume [17, 18]. A lack of power may, of course, explain these negative results. Note also that these trials were not intended to achieve control of PaCO_2_, which may have contributed to the deleterious effects of hypercapnic acidosis in the study arms using reduced tidal volume. Although the clinical evidence is not easy to demonstrate, hypercapnia has unquestionable side effects [19], like increased pulmonary vascular resistance, which can worsen prognosis. In 2000, the ARMA study run by the NHLBI ARDS Network in the USA yielded key data comparing a pulmonary protection strategy using “low” tidal volume, on average 6 mL/kg PBW, a plateau pressure limited to 30 cmH_2_O, and a respiratory rate up to 35 breaths/min, with a non-protection strategy using a tidal volume of 12 mL/kg PBW [20]. The use of PBW calculated as a function of sex and height was an important innovation in adapting tidal volume to the expected lung volume. In this study, increased respiratory rate leading to low-volume ventilation was associated with only a minimal increase in PaCO_2_, a result that may have contributed to the benefits of this treatment arm. A 25% reduction in the relative risk of mortality was observed, i.e., a 30–40% decrease in overall mortality. This study had an enormous impact on clinical practice. It was not the first to use low volumes successfully, that accolade falls to the two-center study by Amato et al., but low tidal volume was combined with higher PEEP, the idea being to reduce driving pressure [21]. Other studies using the same approach as Amato et al. found a similar reduction in mortality [22]. Meta-analyses of tidal volume reduction have often included rather heterogenous studies [23]. The most recent included seven randomized trials in 1481 patients [24] and concluded that lower mortality was associated with low-volume ventilation in primary analysis (hazard ratio 0.80 [0.66, 0.98]) and found a significant relation between tidal volume reduction and the mortality reduction effect. However, when the studies that combined high PEEP and low volumes were excluded, the effect of reduced tidal volume was just a non-significant trend (0.87 [0.70, 1.08]). According to the authors, this suggests, but does not prove, that reduced tidal volumes significantly decrease mortality during ARDS. In an observational study, 11,558 ventilation parameters were available for 482 ARDS patients identified prospectively [25]. The authors compared the patients with volumes of 6.5 mL/kg PBW or less, upon admission, with patients with volumes > 6.5 mL/kg PBW (68% of patients), and found that, after adjustment for known confounding factors, an increase of 1 mL/kg PBW in the settings of the initial volume was associated with a 23% increase in risk of death in intensive care (hazard ratio, 1.23; 95% confidence interval, 1.06–1.44; *p* = 0.008) [25]. A secondary increase in tidal volume was also associated with an increase in mortality risk, but the mortality risk of too high a first tidal volume was higher than the effect of the following volumes [25]. In the LUNG SAFE study [2], tidal volume did not seem to be a significant factor in mortality. However, the volume range was limited [26], which suggests that a “certain degree” of pulmonary protection is used very frequently, but in very few patients with tidal volumes above 10 or below 6 mL/kg. There was no difference in survival in the patients whose tidal volume was equal to or greater than the median value of 7.1 mL/kg PBW [26]. In addition, the use of lower tidal volumes in patients with severe ARDS may involve potentially confounding effects, which are difficult to analyze completely in purely observational data [26]. In all analyses, however, the pressures (peak pressure, plateau pressure, driving pressure, and PEEP) carried more significant weight than tidal volume in the prognosis [26].

Plateau pressure

**R2.2.1 – Once tidal volume is set to around 6** **mL/kg PBW, plateau pressure should be monitored continuously and should not exceed 30 cmH**_**2**_**O to reduce mortality.**

**GRADE 1** **+, STRONG AGREEMENT**

**R2.2.2 - The experts suggest that tidal volume should not be increased when the plateau pressure is well below 30 cmH**_**2**_ **O, except in cases of marked, persistent hypercapnia despite reduction of instrumental dead space and increase of respiratory rate.**

**EXPERT OPINION**

**Rationale:**

Tidal volume, plateau pressure, and driving pressure are closely related (static compliance = tidal volume/plateau pressure-total PEEP) and all participate in VILI. Mechanical ventilation should limit VILI, thereby limiting mortality. Even if VILI was initially observed on application of a high plateau pressure with a high tidal volume [16], there is less lung injury with the same high plateau pressure when the tidal volume is reduced by means of thoracic stiffness [13], a situation encountered in the very obese.

The LUNG SAFE study reported that plateau pressure was not monitored in 60% of ventilated ARDS patients and that a non-negligible proportion of patients, although ventilated with a tidal volume below 8 mL/kg PBW, had a plateau pressure above 30 cmH_2_O, especially those with moderate to severe ARDS [2]. An ancillary study of LUNG SAFE has shown that plateau pressure, which can be modified by the intensivist, is strongly and positively correlated with mortality [26]. A high plateau pressure is an independent mortality risk factor, as it reflects either great severity (associated with poor lung compliance) or inadequate mechanical ventilation [27].

The only way to monitor plateau pressure routinely is to ventilate the patient with an end-inspiratory pause, which should not be too long, so as to facilitate any increase in respiratory rate, or too short, so that the respirator can measure the pressure. A pause of 0.2–0.3 s should be used routinely when adjusting the ventilator.

In a given patient, plateau pressure is an imperfect reflection of lung distension [28]. This is particularly so in patients with abnormal compliance of the chest wall, and in some obese patients. The relation between plateau pressure and mortality or the risk of barotrauma is less clear in these patients [29], which may suggest tolerance of plateau pressure a little above 30 cmH_2_O, provided that the tidal volume is reduced to limit VILI [13]. In all cases, plateau pressure is no longer associated with barotrauma when it is kept below 30 cmH_2_O.

Five controlled and randomized studies compared a strategy of low tidal volume and limited plateau pressure with a strategy using higher tidal volume and plateau pressure [17, 18, 20, 21, 30]. A significant decrease in mortality in the group with limited volume and pressure was observed only in the 2 studies [20, 21] where difference in plateau pressure was particularly large between the 2 strategies tested. When these 5 studies are pooled, there is a strong relation between plateau pressure and mortality [31]. In a recent study in 478 patients, a threshold plateau pressure of 29 cmH_2_O was identified beyond which hospital mortality increased [32]. Even in patients ventilated with a driving pressure below 19 cmH_2_O, a plateau pressure strictly below 30 cmH_2_O would enable a significant reduction in mortality, a greater effect than that of a driving pressure below 19 cmH_2_O when the plateau pressure is already below 30 cmH_2_O [32]. These results were validated in the same study in a different cohort of 300 patients [32].

Driving pressure

**R2.3 – Available data do not allow a recommendation to be made regarding respirator settings based solely on limitation of driving pressure. This limitation can be envisaged as a complement to limitation of plateau pressure in some special instances.**

**NO RECOMMENDATION**

**Rationale:**

One study retrospectively evaluated the influence of driving pressure on prognosis by means of a complex statistical analysis of nine randomized controlled studies of ventilation strategy (comparison of different values of tidal volume and PEEP, during ARDS) [33]. The authors concluded that driving pressure was the best predictor of mortality in these studies. Nonetheless, as the authors themselves acknowledge, this was a retrospective study of studies whose main aim was not to examine the usefulness of driving pressure. No randomized study has since corroborated the value of limiting driving pressure. In contrast, the results of the observational study LUNG SAFE [2, 26] showed no obvious superiority of driving pressure over plateau pressure as a predictor of the risk of mortality. The same was true when the data of two studies showing improved survival during ARDS (by neuromuscular block and by prone positioning) were combined [34]. Prudence regarding the role of driving pressure is advised, and other studies have even yielded some concerns regarding the validity of this physiological concept. Unlike plateau pressure, which translates dynamic and static lung distension, driving pressure translates dynamic distension. A randomized controlled study of PEEP [35] (which showed that a “higher PEEP” was associated with higher mortality) seems to call into question the predictive value of driving pressure. Indeed, plateau pressure was lower in the group with lower mortality, whereas driving pressure was lower in the group with higher mortality [35].

Analysis of a series of mechanically ventilated ARDS patients presenting acute cor pulmonale [36] suggests that when the plateau pressure is kept sufficiently low (< 27 cmH_2_O), driving pressure is predictive of cor pulmonale and of mortality. A randomized study designed to demonstrate the predictive value of driving pressure should therefore limit plateau pressure to less than 30 cmH_2_O or even 28 cmH_2_O in the two groups. Given also that tidal volume should be limited to 6 mL/kg, PEEP is the only ventilator setting that would change. This would therefore amount to comparing two levels of PEEP during ventilation with limited plateau pressure. This is exactly what the EXPRESS study did, and its results were negative [37].

In practical terms, it would be best first to measure and limit plateau pressure, an approach which the LUNG SAFE study [2] has clearly shown is insufficiently used. It is only after limiting plateau pressure sufficiently that we can envisage limiting driving pressure in cases when severely altered lung compliance mandates use of insufficient PEEP to ensure correct oxygenation (for example, in cases when a PEEP of 6–8 cmH_2_O and a tidal volume of 6 mL/kg would generate a plateau pressure of about 30 cmH_2_O in a patient remaining hypoxemic). In this case, it can be useful to reduce driving pressure by further limiting tidal volume, while increasing PEEP, if this maneuver is well tolerated hemodynamically.

### Area 3: Alveolar recruitment

Positive end-expiratory pressure

**R3.1.1 – PEEP is an essential component of the management of ARDS and the experts suggest using a value above 5 cmH**_**2**_ **O in all patients presenting with ARDS.**

**EXPERT OPINION**

**R3.1.2 – High PEEP should probably be used in patients with moderate or severe ARDS, but not in patients with mild ARDS.**

**GRADE 2** **+, STRONG AGREEMENT**

**R3.1.3 – The experts suggest reserving high PEEP for patients in whom it improves oxygenation without marked deterioration of respiratory system compliance or hemodynamic status. PEEP settings should be individualized.**

**EXPERT OPINION**

**Rationale:**

PEEP is an integral part of the protective ventilation strategy. The expected beneficial effect of high PEEP is optimized alveolar recruitment, which, on the one hand, decreases the intrapulmonary shunt, thus improving arterial oxygenation, and, on the other hand, decreases the amount of lung tissue exposed to alveolar opening-closing, thus reducing the risk of VILI [38, 39]. Conversely, the deleterious effects of high PEEP are increased end-inspiratory lung volume, hence increased risk of volutrauma [13], hemodynamic worsening linked to a decrease in preload, and above all to an increase in right ventricular afterload [40, 41]. When total PEEP is constant, the effects of intrinsic PEEP are, during ARDS, identical to those of external PEEP [42, 43].

The extent of the beneficial and deleterious effects of high PEEP varies greatly from one patient to another and cannot be predicted from the simple clinical data available at the bedside. However, studies using chest CT scans have shown that, on average, the amount of potentially recruitable lung tissue with high PEEP is greater when the PaO_2_/FiO_2_ ratio measured with a low PEEP (5 cmH_2_O) is low [44, 45].

A post hoc analysis of 2 randomized trials shows that, in patients in whom randomization led to increased PEEP, in-hospital mortality was lower for greater increases in the PaO_2_/FiO_2_ ratio after increase of PEEP [46].

Individually, the effect of high PEEP in terms of recruitment cannot be assessed from changes in respiratory system compliance [45, 47]. No blood gas or respiratory mechanics parameter easily available at the bedside allows quantification of the risk of volutrauma induced by the use of high PEEP. On average, the levels of PEEP used in randomized trials comparing “high” and “moderate” PEEP were, respectively, 15.1 ± 3.6 cmH_2_O and 9.1 ± 2.7 cmH_2_O [24]. Thus, 12 cmH_2_O can be considered as the threshold above which PEEP can be qualified as high.

No significant difference in mortality was found in any of the 3 large randomized trials that compared the impact of high and moderate PEEP in ARDS patients ventilated with a tidal volume of 6 mL/kg PBW [37, 48, 49]. A meta-analysis of the individual data from patients included in these 3 trials showed that high PEEP was associated with a significant 5% reduction in hospital mortality in patients with moderate or severe ARDS (34.1% vs. 39.1%, *p* < .05), whereas it was associated with greater mortality (27.2% vs. 19.4%, *p* = .07) in patients with mild ARDS [50].

In patients with moderate or severe ARDS, individualized PEEP setting using end-expiratory transpulmonary pressure did not result in a decrease in mortality compared to PEEP set using a PEEP/FiO_2_ scale [51].

High-frequency oscillation ventilation

**R3.2. – High-frequency oscillation ventilation should not be used in ARDS patients.**

**GRADE 1** **−, STRONG AGREEMENT**

**Rationale:**

High-frequency oscillation ventilation (HFOV) is an unconventional mode of ventilation proposed to improve gas exchange while protecting against VILI using a tidal volume below or equal to the anatomical dead space [52]. Continuous gas flow creates a continuous distending airway pressure (cP_aw_) so as to recruit the pulmonary parenchyma, whereas the sinusoidal oscillations of a membrane at a high respiratory rate (3–8 Hz) generate tidal volume. The gas flow and the inflation of a balloon valve allow adjustment of cP_aw_, which determines oxygenation proportionally. Tidal volume increases with the amplitude of the membrane movements and decreases when the frequency increases, which explains why CO_2_ removal is inversely proportional to the frequency used.

Numerous physiological studies have suggested that HFOV is useful in the management of ARDS. Thanks to exchange mechanisms distinct from simple exchange by convection [53], HFOV enables a greater reduction in tidal volume and decreases the amplitude of cyclic variations in transpulmonary pressure, thus allowing the use of a high cP_aw_ so as to optimize lung recruitment. By increasing the proportion of parenchyma ventilated, the recruitment induced in HFOV may reduce lung stress and strain, reduce the sheer stress associated with the cyclic opening and closing of unstable alveoli, and limit VILI. Hence, the ventilation characteristics in HFOV make it theoretically ideal in terms of lung protection [52, 54].

Several clinical studies have reported that HFOV improves oxygenation in adults with ARDS and refractory hypoxemia in conventional ventilation [55–58]. Three randomized studies reported a tendency to decreased mortality when HFOV was used as an initial mode of ventilation in 58, 148 and 125 ARDS patients, respectively [59–61]. However, the use of excessive tidal volume in the control group limits the value of these studies, which do not allow recommendation of HFOV as the main mode of ventilation for ARDS. Recently, 2 large randomized trials found no benefit of HFOV compared with conventional mechanical ventilation with tidal volume = 6 mL/kg, limitation of plateau pressure, and PEEP adapted as a function of ARDS severity [62, 63]. In the OSCILLATE study, an aggressive recruitment strategy in HFOV was even associated with a significant rise in mortality [62]. It is possible that the use of a high cP_aw_ induced overdistension without increasing aeration in alveolar collapse or flooding, in particular in patients presenting heterogeneous lesions and a limited percentage of recruitable parenchyma. The use of high pressures may also have induced an increase in right ventricular afterload, right ventricular insufficiency [64], and hemodynamic instability requiring higher doses of vasopressors [62]. With a cP_aw_ titration strategy based on the mean alveolar pressure used before the initiation of HFOV and the response in terms of oxygenation, Young et al. found no difference in mortality in the OSCAR study when HFOV was compared with conventional mechanical ventilation in ARDS patients [63]. In 2016, the LUNG SAFE study revealed that HFOV was used in 1.2% of ARDS patients [2].

Several systematic meta-analyses of 5 randomized studies evaluated secondary endpoints, such as gas exchange and the incidence of barotrauma [65–68]. They did not show significant improvement in gas exchange or reduction in barotrauma with HFOV. A recent meta-analysis of individual data suggests that HFOV may improve survival in patients with more severe hypoxemia [66]. The ideal modalities for cP_aw_ titration, oscillation frequency, and monitoring of HFOV are poorly defined. In particular, studies are needed to determine whether evaluation of transpulmonary pressure by measurement of esophageal pressure is useful in regulating cP_aw_, improving lung recruitment, and avoiding overdistension [69]. Pending the results of an ongoing study that is testing this hypothesis (Clinical Trials.gov NCT02342756), HFOV should be limited to clinical trials in patients with severe ARDS in whom conventional mechanical ventilation has failed despite prone positioning, and should be performed in centers with considerable experience of HFOV.

Recruitment maneuvers

**R3.3 – Recruitment maneuvers should probably not be used routinely in ARDS patients.**

**GRADE 2** **−, STRONG AGREEMENT**

**Rationale:**

In cases of clear derecruitment (endotracheal aspiration, accidental or planned disconnection, intubation…), use can be made of a careful recruitment maneuver. If hypoxemia is refractory (PaO_2_/FiO_2_ < 100 mmHg) despite optimization of therapy, a recruitment maneuver can be envisaged in the absence of contraindication.

There is no preferred recruitment maneuver. The recommended procedure should last no longer than 10–20 s, and the airway pressure should not exceed 30–40 cmH_2_O. The recruitment maneuver should be performed with care and should be interrupted if hemodynamic safety is poor.

ARDS patients frequently present pulmonary atelectasis, which decreases the ventilated lung volume, worsens hypoxemia, and increases VILI [70]. The recruitment maneuver, by the application of a transiently high airway pressure, is intended to expand the collapsed lung so as to increase the number of alveolar units participating in gas exchange [71].

Several different maneuvers are used, such as the application of a continuous positive pressure (30–40 cmH_2_O) for 30–40 s, or the progressive increase of PEEP at constant driving pressure, or the progressive increase of driving pressure at constant PEEP [72–74]. Recruitment maneuvers improve oxygenation and dynamic compliance [75–77]. By application of a high intra-alveolar pressure, they may run the risk of barotrauma related to overdistension of alveoli. By increasing intrathoracic pressure, they can reduce peripheral venous return and right ventricular preload, thereby inducing or worsening hemodynamic instability (particularly in hypovolemic patients) [73].

Recruitment maneuvers were evaluated in 8 controlled randomized studies [21, 35, 49, 78–82] in a total of 2735 patients between 1998 and 2018. The nature of the maneuvers used and the target airway pressures during the maneuver differed substantially between studies. Four of the 8 studies recommended application of a continuous positive airway pressure of 40 cmH_2_O for 40 s [21, 49, 80, 82]. Seven of the 8 studies combined the recruitment maneuver with application of a high PEEP, with the aim of keeping recruited alveoli open [21, 35, 49, 78–81].

In the 8 studies, the use of recruitment maneuvers was not significantly associated with a reduction in mortality at day 28 (RR = 0.89—95% CI [0.89–1.07]). In the only study without co-intervention, recruitment maneuvers were associated with reduced mortality (110 patients, RR = 0.81—95% CI [0.69–0.95]). In each of the 7 studies (2625 patients) that gave the PaO_2_/FiO_2_ ratio at day 1, it was significantly higher in the patients managed using a recruitment maneuver (average of the averages: 205.9 mmHg vs. 158.3 mmHg) [21, 35, 49, 78–81]. This improvement in PaO_2_/FiO_2_ persisted till day 77 (average of the averages: 231.2 mmHg vs. 195.1 mmHg) in the same 7 studies (2625 patients) [21, 35, 49, 78–81]. There was no evidence that a recruitment maneuver increased the risk of barotrauma (RR = 1.25—95% CI [0.93–1.67]) in 6 studies [21, 35, 49, 78, 80, 81]. In contrast, there was significantly greater worsening of hemodynamic status (RR = 1.22—95% CI [1.04–1.45]) [35, 81].

There is as yet no proven optimal recruitment maneuver, notably to minimize hemodynamic risk and the risk of barotrauma, while preserving efficacy in terms of lung oxygenation. A recent study [80] opens up a new possibility by adapting the indication for a recruitment maneuver to the CT scan findings (diffuse or focal) in ARDS. The search for a better target population among ARDS patients could provide new information concerning the effect of recruitment maneuvers on mortality.

### Area 4: Spontaneous ventilation

Early and short neuromuscular blockade

**R4.1 – A neuromuscular blocking agent should probably be considered in ARDS patients with a PaO**_**2**_**/FiO**_**2**_ **ratio** **<** **150** **mmHg to reduce mortality. The neuromuscular blocking agent should be administered by continuous infusion early (within 48** **h after the start of ARDS), for no more than 48** **h, with at least daily evaluation.**

**GRADE 2** **+, STRONG AGREEMENT**

**Rationale:**

Three randomized trials tested the effect of the addition of a neuromuscular blocking agent to deep sedation at the initial phase of ARDS [83–85]. The primary outcome of only one of these trials was mortality [85]. A randomized open trial (Reevaluation of Systemic Early Neuromuscular Blockade [ROSE]) methodologically slightly different is currently being analyzed [86]. The ACURASYS study [85] included 339 patients presenting with ARDS with a progression of less than 48 h and with a PaO_2_/FiO_2_ ratio < 150 mmHg, PEEP ≥ 5 cmH_2_O, and tidal volume from 6 to 8 mL/kg PBW in a double-blind, placebo-controlled multicenter study. Patients were included after optimizing invasive mechanical ventilation. Cisatracurium besylate was the neuromuscular blocking agent used. The 90-day mortality did not differ between patients treated with cisatracurium and those treated with placebo (31.6% vs. 40.7%, respectively; *p* = 0.08). However, the hazard ratio for 90-day mortality in the cisatracurium group was 0.68 (95% CI 0.48–0.98; *p* = 0.04), after adjustment for the PaO_2_/FiO_2_ ratio, plateau pressure, and the Simplified Acute Physiology II score at inclusion [85]. There was improved survival in the patients with a PaO_2_/FiO_2_ ratio < 120 mmHg. There were more days alive and free of mechanical ventilation at day 90 in the cisatracurium group (HR 1.41; *p* = 0.01), and there was no between-group difference in the rate of intensive care unit-acquired paresis [85].

Oxygenation (PaO_2_/FiO_2_) increases when neuromuscular blocking agents are used in ARDS patients [83, 84, 87, 88].

In a retrospective study, cisatracurium was not superior to atracurium [89]. In contrast, the duration of mechanical ventilation and the length of ICU stay were slightly but significantly shorter in patients with or at risk of ARDS who were treated with cisatracurium, compared with those treated with vecuronium [90].

The depth of neuromuscular block required is unknown. The ACURASYS study used high dosages of cisatracurium (37 mg/h) [85].

Neuromuscular blocking agents could have beneficial effects in limiting expiratory efforts and Pendelluft effect, and in increasing expiratory transpulmonary pressure [88].

Early spontaneous ventilation

**R4.2.1 – Available data do not allow a recommendation to be made regarding a strategy of routine spontaneous ventilation in the acute phase of ARDS.**

**NO RECOMMENDATION**

**R4.2.2 – After the acute phase of ARDS, the experts suggest that ventilation with a pressure mode allowing spontaneous ventilation can be used when ensuring that the tidal volume generated is close to 6** **mL/kg PBW and does not exceed 8** **mL/kg PBW.**

**EXPERT OPINION**

**Rationale:**

The term spontaneous breathing refers to the activity of the respiratory muscles, which is responsible for spontaneous ventilation (SV) in the ventilated patient. The importance of SV depends on the intensity of the breathing efforts and on the impedance of the respiratory system [91]. Spontaneous breathing efforts are present in most ventilated patients, except for those in so-called controlled ventilation who are paralyzed and/or deeply sedated. Spontaneous breathing has very different consequences depending on the mode of ventilation used [92]. During assisted controlled ventilation (either pressure or volume regulated), breathing efforts tend to increase minute ventilation by triggering (via the inspiratory trigger) the ventilator. In this setting, tidal volume can worsen lung injury (concept of patient self-inflicted lung injury) [93]. The interaction can be more complex and responsible for patient-ventilator asynchrony, which in some cases increases tidal volume and may worsen the prognosis [94, 95]. Asynchrony can be limited by adapting the ventilator settings or abolished by neuromuscular blocking agents administration.

With specific pressure-controlled ventilation modes, which does not offer the possibility of inspiratory synchronization (absence of trigger as in airway pressure release ventilation or APRV), breathing efforts generate SV, which is superimposed on mechanical ventilation cycles [91]. Spontaneous breathing efforts have beneficial effects (improved oxygenation, alveolar recruitment, prevention of ventilation-induced diaphragmatic lesions), which should be balanced with deleterious effects (increase in transpulmonary pressure and tidal volume, pendelluft, increased transvascular pressure of the vessels in the lung interstitium, and risk of pulmonary edema) [91]. The benefit-risk balance depends on the severity of respiratory disease and on the level of SV [91]. SV above 30 or 50% of the total minute ventilation is possibly harmful. If the ventilation defined by the ventilator settings is increased and/or if sedation is too deep, SV tends to decline. Conversely, SV increases if the ventilation set on the ventilator is insufficient and/or if sedation is insufficient or in cases of metabolic acidosis [92].

SV can be modulated by sedation and by the level of ventilation delivered by the ventilator.

Nonsynchronized pressure-controlled ventilation (like APRV) favors SV by limiting the asynchrony observed with pressure- or volume-controlled assisted ventilation. SV associated with nonsynchronized pressure-controlled ventilation (like APRV) is associated with increased respiratory effort, which can be detected by variations in airway occlusion pressure.

The beneficial effect of SV on oxygenation and respiratory mechanics has been demonstrated in animal models and confirmed by clinical studies in small numbers of patients. A single-center randomized study comparing SV in APRV versus pressure-controlled ventilation (sedation and neuromuscular block) in 30 mechanically ventilated patients with multiple trauma showed a favorable effect of SV on gas exchange, respiratory mechanics, and the duration of ventilation [96]. The sedation strategy, the large between-group difference in ventilation modalities, and the small number of patients prevent conclusions being drawn regarding the benefit of SV. These methodological obstacles are found in most studies assessing the benefit of SV.

In a recent, randomized single-center trial in 138 patients ventilated for at least 48 h with a PaO_2_/FiO_2_ ratio < 250 mmHg, a protective ventilation strategy (6 mL/kg PBW, plateau pressure < 30 cmH_2_O, PEEP guided by the PEEP-FiO_2_ table according to the ARDSNet Protocol) was compared with APRV (tidal volume 6 mL/kg PBW, plateau pressure < 30 cmH_2_O, PEEP 5 cmH_2_O) designed to encourage SV [97]. The sedation strategy was common to the two study arms. The number of days without ventilation at day 28 (principal endpoint) was significantly greater in the APRV arm. Likewise, compliance and oxygenation parameters were significantly improved in APRV, while there was less sedation requirement [97]. Tidal volume and driving pressure were comparable in the two arms, while PEEP and plateau pressure were significantly lower in APRV [97]. The main limitations of this study are that it was single-center, there were few patients, and the experience of the “respiratory therapists” who adjusted the APRV settings, which are hard to master [97]. Nonetheless, this study shows the feasibility of a strategy designed to reach modest levels of SV (approximately 30% of the minute ventilation). The complications were not more frequent in the APRV arm, in which the incidence of pneumothorax was low (4.2%) [97].

A nonsynchronized mode (like APRV) was compared (crossover, randomized physiological study) with completely or partially synchronized pressure-controlled ventilation [98]. Tidal volume and transpulmonary pressure were significantly lower in cases of nonsynchronization, whereas SV was associated with increased breathing efforts, which could be detected by monitoring airway occlusion pressure [98].

A randomized, controlled multicenter trial has compared the impact of ventilation that systematically encourages SV with assisted controlled ventilation, for a given strategy in the settings of tidal volume, end-inspiratory pressure, PEEP, sedation, weaning PEEP, and weaning ventilation. This trial (BiRDS) finished after the inclusion of 700 patients and the results are pending (www.clinicaltrials.gov NCT01862016). The study protocol enabled adaptation of the level of sedation and ventilation so as to achieve the aim of SV.

### Area 5: Prone positioning

**R5.1 – Prone positioning should be used in ARDS patients with PaO**_**2**_**/FIO**_**2**_ **ratio** **<** **150** **mmHg to reduce mortality. Sessions of at least 16 consecutive hours should be performed.**

**GRADE 1** **+, STRONG AGREEMENT**

**Rationale:**

The use of prone positioning (PP) during ARDS has been studied in 8 randomized controlled trials, 5 of which were large [10, 45, 99–101] and 3 smaller [102–104]. The most recent meta-analysis concluded that there was no statistically significant difference in mortality between the PP group and the supine position group [105]. This meta-analysis included 3 sensitivity analyses on the role of protective ventilation, the duration of PP, and the severity of hypoxemia at the time of inclusion. When the trial protocol provided for protective mechanical ventilation, there was a non-significant reduction in mortality in favor of PP [105]. This reduction in mortality was significant for PP lasting longer than 12 h, but it not for shorter PP sessions [105]. The reduction in mortality in favor of PP was significant for the most hypoxemic patients with moderate to severe ARDS, but was not significant for less hypoxemic patients (mild ARDS).

The PROSEVA study [10] done in 27 intensive care units showed a significant reduction in mortality in ARDS patients included after a 12- to 24-h stabilization period with a PaO_2_/FIO_2_ ratio < 150 mmHg associated with PEEP of at least 5 cmH_2_O, an FIO_2_ of at least 60%, and tidal volume of 6 mL/kg PBW. This confirmed a previous meta-analysis on individual data [106]. In the PROSEVA trial PP group, the patients had on average 4 PP sessions of 17 consecutive hours (the protocol planned sessions of at least 16 h). PP was continued even in the absence of improved oxygenation.

PP is inexpensive and simple to implement. Optimization of the safety of PP requires that each department has a written procedure and specific training of nursing teams.

### Area 6: Extracorporeal gas exchange

Venovenous extracorporeal membrane oxygenation

**R6.1 – Venovenous extracorporeal membrane oxygenation (ECMO) should probably be considered in cases of severe ARDS with PaO**_**2**_**/FiO**_**2**_ **<** **80** **mmHg and/or when mechanical ventilation becomes dangerous because of the increase in plateau pressure and despite optimization of ARDS management including high PEEP, neuromuscular blocking agents, and prone positioning. The decision to use ECMO should be evaluated early by means of contact with an expert center.**

**GRADE 2** **+, STRONG AGREEMENT**

**Rationale**:

Few studies have assessed the efficacy of ECMO in ARDS. The multicenter CESAR trial [107] randomized 180 patients to transfer to an ECMO center for consideration for ECMO or to conventional ventilatory support. The primary outcome of death and/or severe disability at 6 months was significantly less frequent in the ECMO group, but its interpretation is limited by a large number of control patients who did not receive protective ventilation, and by the fact that 25% of the patients randomized to the transfer and consideration for ECMO group did not actually receive ECMO [107].

Two retrospective case-controlled studies using propensity score matching [108, 109] suggested a benefit of transferring patients with A(H1N1)-related ARDS during the 2009 influenza pandemic to an expert venovenous ECMO referral center.

The randomized EOLIA trial [110] evaluated the effect of early initiation of venovenous ECMO in severe ARDS while avoiding the methodological biases of CESAR. This multicenter trial included 249 patients with severe ARDS on mechanical ventilation for less than 7 days. Patients randomized to the early ECMO group received immediate percutaneous venovenous cannulation while control group patients were managed with protocolized conventional mechanical ventilation. At inclusion, the average PaO_2_/FiO_2_ ratio was 72, the SOFA score was above 10, and 75% of the patients were receiving vasopressors [110]. It should be noted that all control group patients received neuromuscular blocking agents and that 90% of them had prolonged sessions of PP. Sixty-day mortality was 11% lower in the ECMO group (35% versus 46%), though the difference did not reach statistical significance (*p* = 0.09) [110]. In contrast, the risk of treatment failure at day 90 (death in the ECMO group, death or crossover to ECMO in the control group) was significantly higher in the control group [110]. Complications associated with ECMO were infrequent, and fewer cases of stroke were observed in the ECMO group. Salvage ECMO was used in 28% of control patients because of refractory hypoxemia [110]. These patients were extremely ill, and their clinical state deteriorated rapidly in the hours before initiation of ECMO. Their mortality was 57% and 6 required venoarterial ECMO while undergoing cardiopulmonary resuscitation [110].

Although the frequentist analysis of this study is negative in a strictly statistical sense (60-day mortality, 35% vs 46%, *p* = 0.09), a post hoc Bayesian analysis of EOLIA [111] with various assumptions of prior belief and knowledge about ECMO efficacy in ARDS has shown that the posterior probability of a mortality reduction with ECMO as in the EOLIA trial, was very high (between 88 and 99%). Furthermore, the EOLIA trial showed that ECMO was safe when provided in high-volume expert centers [110]. It allows the application of ultraprotective ventilation in which pressures and volumes generated by the respirator are drastically reduced, thus protecting the lung from further ventilation-induced lung injury. The EOLIA trial has also demonstrated the relevance and efficacy of hospital networks to safely retrieve on ECMO the most severely ill patients 24/7 with an ECMO mobile team to an ECMO referral center [110].

Low-flow extracorporeal CO_2_ removal

**R6.2 – Available data do not allow a recommendation to be made concerning the use of low-flow extracorporeal CO**_**2**_ **removal during ARDS.**

**NO RECOMMENDATION**

**Rationale:**

Arteriovenous or venovenous low-flow extracorporeal CO_2_ removal (ECCO_2_R) allows so-called “ultraprotective” ventilation strategies (tidal volume < 6 mL/kg PBW and decrease in plateau and driving pressures and in respiratory rate) during ARDS, by controlling hypercapnia induced by the reduction in minute ventilation. Ten studies tested this approach [112–121], but the overall level of proof is very low. In the only recent randomized controlled trial that included 79 patients, the numbers of ventilator-free days at day 60 were not different between control and ECCO_2_R groups, although a post hoc analysis suggested a benefit of ECCO_2_R for the most hypoxemic patients (PaO_2_/FiO_2_ < 150 mmHg at inclusion) [113].

Observational studies suggest that hypercapnia has an unfavorable prognostic effect in ARDS; it is associated in multivariate analysis with pulmonary vascular and right ventricular dysfunction [36] and with mortality [19]. ECCO_2_R can decrease PaCO_2_ in hypercapnic ARDS patients receiving “conventional” protective ventilation (tidal volume about 6 mL/kg PBW) [112, 115, 116, 122–124] or ultraprotective ventilation [117, 120]. Nonetheless, the positive effect of the control of hypercapnia on morbidity and mortality has yet to be demonstrated in ARDS.

The effect of ECCO_2_R on PaO_2_ in ARDS patients is inconstant, some studies reporting an improvement [119–122] and many others no significant effect [112, 114, 115, 117, 118, 124, 125]. Because ECCO_2_R only provides marginal blood oxygenation, venovenous ECMO is required in the most hypoxemic patients. Lastly, ECCO_2_R is associated with a wide range of complications (bleeding, thrombosis, and infections) that should be balanced against its potential benefits [126].

### Area 7: Inhaled nitric oxide

**R7.1 – The experts suggest that inhaled nitric oxide can be used in cases of ARDS with deep hypoxemia despite the implementation of a protective ventilation strategy and prone positioning, and before envisaging use of venovenous ECMO.**

**EXPERT OPINION**

**Rationale:**

Initially considered as a pollutant, nitric oxide (NO) is a ubiquitous, odorless and colorless gas whose properties were demonstrated by Furchgott, Ignarro, Murad, and Moncada in work that was rewarded by a Nobel Prize [127]. Produced by endothelial cells, NO induces vasodilation by increasing the level of cyclic GMP in smooth muscle cells. Depending on its concentration, NO, in addition to its vasomotor properties, produces numerous potentially interesting pro- or anti-inflammatory effects in the setting of ARDS [128]. Notably, it attenuates leukocyte activation and inflammatory responses, reduces platelet aggregation, has a bronchodilator effect, and facilitates the production of surfactant.

When inhaled, NO diffuses into ventilated areas where it induces vasodilation before rapidly binding to hemoglobin by a reaction with the ferrous and ferric ion of heme to form nitrosylated hemoglobin [128]. By reacting with oxyhemoglobin, the predominant form in the lung, NO forms methemoglobin and nitrates and does not result in systemic vasodilation. Approximately 70% of inhaled NO (iNO) is eliminated in the form of nitrate in urine [129]. iNO is a selective pulmonary arterial vasodilator likely to improve gas exchange by reducing the shunt and to control pulmonary arterial hypertension and right ventricular insufficiency, which has an unfavorable prognosis in ARDS [130, 131]. In addition, its effects on platelets and leukocytes could prove of therapeutic value in ARDS.

Inhalation of NO dilates the pulmonary vessels in ventilated areas and improves the ventilation-perfusion ratio by preferentially redistributing the blood flow to these areas. Eleven randomized trials report an improvement in the PaO_2_/FiO_2_ ratio after 24 h of treatment [132]. However, this improvement is transient and only an analysis based on 4 trials indicates improvement that persists after 96 h of treatment [132]. Note that the response is greater if there is pulmonary arterial hypertension, that the concentrations likely to improve oxygenation are generally below 5 parts per million (ppm) [133], and that concentrations above 10 ppm are sometimes associated with a worsening of the PaO_2_/FiO_2_ ratio, possibly because of the diffusion of NO into unventilated areas [134].

To date, 8 randomized studies in a total of 1025 adults with ARDS, including at least 10 treated with iNO, evaluated the impact of this treatment on mortality [133, 135–140]. None of these studies found significant improvement in survival at 28 days or long term. Analysis of available randomized studies reveals that iNO does not change the duration of mechanical ventilation, the time spent in intensive care, or the onset of barotrauma complications. Published between 1997 and 2004, most of these studies have a relatively modest risk of bias, but they suffer from a certain number of methodological problems that complicate the interpretation. Most of these studies lack power and evaluate the response of heterogenous patients in terms of the etiology of ARDS. The modalities of administration (concentration, duration, evaluation of the response, weaning) and of monitoring were insufficiently defined and varied greatly from one study to another. Also, these studies were conducted before the generalization of protective ventilation strategies for ARDS. In the most recent study, in 385 patients, the tidal volume used in the 2 groups was 10 mL/kg [139]. Compliance with a protective ventilation strategy is not reported in any study, and there were no protocols for mechanical ventilation weaning or for optimization of sedation in these studies. It is therefore difficult to draw definitive conclusions as to any benefit of iNO in ARDS.

Given a quite favorable benefit-risk ratio, the physiological effects of iNO on the reduction in the intrapulmonary shunt, and the improvement of gas exchange, right ventricular performance, and cardiac flow may justify its use in severe ARDS when PP and optimization of mechanical ventilation do not correct hypoxemia. Data from physiological studies and the main clinical trials suggest that iNO has a good safety profile and that its potential adverse effects, notably methemoglobinemia, inhibition of platelet aggregation, and systemic vasodilation, are not clinically significant if a few precautions are observed [135, 141–143]. In the presence of oxygen, NO is transformed into nitrite (NO_2_) and then nitrate (NO_3_). However, if inhaled with a high FiO_2_, NO together with reactive oxygen species can form potentially toxic molecules, in particular peroxynitrite (ONOO^−^) [141]. NO can also bind to amino acids such as tyrosine and engender posttranslational changes in proteins, such as nitrosation, nitrosylation, and nitration. Furthermore, a risk of renal toxicity has been described in a clinical trial [136] and in a recent meta-analysis [132]. A systematic review of trials reveals that the risk of renal toxicity seems to be limited to ARDS patients exposed to high iNO concentrations for prolonged periods [144]. To limit the risk of complications with iNO, it is appropriate to: (1) minimize exposure by using systems of administration that enable inhalation synchronized with inspiratory flow and precise monitoring of the concentrations of NO and NOx, (2) use the minimum effective concentration to improve the PaO_2_/FiO_2_ ratio and not maintain iNO in a nonresponsive patient, (3) reevaluate the response and the required dosage daily. In cases of prolonged use, methemoglobinemia should also be monitored. Lastly, weaning from iNO should be progressive so as to limit the risk of a sudden increase in pulmonary arterial pressure.

## Data Availability

Not applicable.

## Abbreviations

APRV: airway pressure release ventilation
ARDS: acute respiratory distress syndrome
cP_aw_: continuous distending airway pressure
ECMO: extracorporeal membrane oxygenation
ECCO_2_R: extracorporeal CO_2_ removal
GRADE: Grade of Recommendation Assessment, Development and Evaluation
HFOV: high-frequency oscillation ventilation
ICU: intensive care unit
NO: nitric oxide
PBW: predicted body weight
PEEP: positive end-expiratory pressure
PICO: Patient Intervention Comparison Outcome
PP: prone position
PPM: parts per million
SRLF: Société de Réanimation de Langue Française
SV: spontaneous ventilation
VILI: ventilator-induced lung injury
